# Antibacterial Effect of Two Nano Zinc Oxide Gel Preparations Compared to Calcium Hydroxide and Chlorhexidine Mixture 

**DOI:** 10.22037/iej.v13i3.19866

**Published:** 2018

**Authors:** Mohammad Samiei, Ali Torab, Oldouz Hosseini, Teymur Abbasi, Amir Ardalan Abdollahi, Baharak Divband

**Affiliations:** a *Department of Endodontics, Dental School, Tabriz University of Medical Sciences, Tabriz, Iran; *; b * Department of Prosthodontics, Dental and Periodontal Research Center, Dental School, Tabriz University of Medical Sciences, Tabriz, Iran* *; *; c * Dental and Periodontal Research Center, Dental School, Tabriz University of Medical Sciences, Tabriz, Iran* *; *; d * Dental School Hospital, Tabriz University of Medical Sciences, Tabriz, Iran* *; *; e * Department of Endodontics, Dental School, Urmia University of Medical Sciences, Urmia, Iran* *; *; f * Drug Applied Research Center, Tabriz University of Medical Sciences, Tabriz, Iran*

**Keywords:** Calcium Hydroxide, Chlorhexidine, *E. faecalis*, Nanoparticles, Zinc Oxide

## Abstract

**Introduction:**

The aim of this study was to compare the antibacterial effects of two gels containing zinc oxide and zinc oxide/silver nanoparticles and a mixture of calcium hydroxide and 0.12% chlorhexidine as intracanal medicaments in root canals contaminated with *Enterococcus faecalis* (*E. faecalis*) at different time intervals.

**Methods and Materials:**

After preparation and culturing of *E. faecalis* in 132 single root teeth, the initial count of bacteria was performed. Then, different materials as intracanal medicaments were used in periods of 3, 7 and 14 days (group 1: calcium hydroxide with 0.12% CHX paste; group 2 zinc oxide nanoparticles gel; group 3: zinc oxide/silver nanoparticles gel; group 4: normal saline as the control group). After the specified time, intracanal medicament was removed and the final count of bacteria was performed. Antibacterial effect of materials was counted by measuring the percentage reduction in the colony counts (RCC). Data were analyzed using the descriptive statistics (Mean±SD) and multi-factorial analysis of variance (by taking into account the effect of the time factor on the dependent variable).

**Results:**

There were no statistically significant differences among mean RCC of different time intervals in each group (*P*=0.09). However, the differences in mean RCC of different dressing materials were significant (*P*<0.001). The effect of interaction between time and materials was significant (*P*=0.015). Comparison of the antibacterial effects of experimental agents at different time intervals showed that the mean RCC in group 1 was higher than other groups (*P*<0.001). The difference in antibacterial effect between groups 2 and 3 was not significant (*P*>0.05). The minimum antibacterial effect was observed in group 4 (*P*<0.0001).

**Conclusions:**

The mixture of calcium hydroxide/chlorhexidine as an intracanal medicament was more effective than zinc oxide and zinc oxide/silver nanoparticles gels.

## Introduction

Microorganisms are the etiologic factors for the pulp and periapical diseases; therefore, the chief aim of endodontic treatment is to eliminate microorganisms from the root canal space [1]. Studies have shown that although mechanical debridement and shaping of the root canal in association with the use of appropriate irrigation solutions decreases the number of microorganisms [2], the results of bacterial cultures of samples, taken from the root canal space; are still positive in many cases [3, 4]. *Enterococcus faecalis* (*E. faecalis*) is one of the resistant bacteria species that remains within the root canal, and its presence in the root canal is associated with an increase in the failure rate of initial endodontic treatment and even retreatment [2]. Some of the reasons for using intracanal medicaments are to eliminate intracanal bacteria, prevent proliferation of bacteria between appointments, create a physiomechanical barrier and accelerate recovery of pathologic periapical tissues [5]. Despite all the researches and discussions over intracanal dressings, still no material is available that can ideally be used to this end. Despite all this, the most commonly used intracanal medicament is calcium hydroxide (Ca (OH)_2_); and also potassium iodine (KI), and chlorhexidine (CHX) [3, 6]. The efficacy of each can be influenced by several factors such as pH, serum proteins, collagen, and dentin among others [6]. For example, the most important problem of calcium hydroxide is neutralization of its alkaline pH due to the buffering capacity of dentin [7]. In addition, the majority of studies have shown that this material does not have any effect on *E. faecalis* [5, 8], questioning the use of this material in its present form [9]. One of the techniques to increase the efficacy of calcium hydroxide is to mix it with chlorhexidine (CHX) [1, 9]. CHX is a broad-spectrum antibacterial agent and studies have shown its effect on *E. faecalis *[6, 9, 10].

A large number of studies have been undertaken in order to introduce a material with better properties, including the use of nanotechnology to fabricate antimicrobial agents. The advantage of the use of nanotechnology is an increase in the surface-to-volume ratio of the materials, which increases the solubility, chemical activity and antibacterial efficacy of these agents as intracanal medicaments [11]. In endodontics, nanoparticles have been used as irrigants, intracanal medicaments or root canal sealers [6, 12]. Some of the materials which have exhibited improved properties with the application of nanotechnology are nanoparticles of zinc oxide and silver. 

The antibacterial mechanism of zinc oxide (ZO) has not been precisely elucidated; however, based on some studies the release of hydrogen peroxide from ZO or binding of zinc to bacterial surfaces is responsible for its antibacterial properties [13]. Studies have shown the greatest effect of ZO nanoparticles on gram-positive bacteria (including *E. faecalis*) compared to gram-negative bacteria [14, 15]; in addition, ZO decreases the adhesion of *E. faecalis* up to 80-95% [16]. On the other hand, incorporation of ZO nanoparticles into other materials used as intracanal medicaments results in an improvement in antibacterial properties of these materials [16]. 

The antibacterial properties of silver depend on silver concentration, release rate and its ability to bind to specific thiol groups containing sulfur and hydrogen in bacterial structures [17]. The results of various studies have shown the superiority of ZO to silver in terms of its biocompatibility [15, 18]. The hypothesis was that ZO and ZO/silver nanoparticles gels was effective same as the mixture of calcium hydroxide/chlorhexidine as an intracanal medicament. Since there was no study evaluating the antibacterial effects of nano particles of ZO and Nano silver/ZO in comparison to common intracanal medicaments, this study aimed to compare the antibacterial effects of gels containing ZO and ZO/silver nanoparticles, and a mixture of calcium hydroxide and 0.12% CXH as intracanal medicaments in root canals contaminated with *E. faecalis* at different time intervals. 

## Materials and Methods

The study was approved by the Research and Ethics Committee of Tabriz University of Medical Sciences. The sample size of this study was calculated according to Madhubala *et al.* [7] using *α*=0.05 and study power of 80%. One hundred thirty two single-rooted human teeth (with one root canal) extracted for orthodontic reasons or due to periodontal problems were used for the purpose of this *in vitro* study. Subsequent to extraction, the teeth were stored in 3% chloramine-T solution at 4^°^C. The teeth were cleaned of all the remaining soft tissues and calculi using mechanical and chemical methods and stored in normal saline solution until used for the purpose of the study. 

Teeth with cracks, developmental abnormalities, immature apices, root fractures, root resorption, root surface caries, deep concavities, calcification and previous endodontic treatment were identified using periapical radiographs and excluded from the study. 

***Preparation of samples***

The tooth crowns were removed at cemento-enamel junction (CEJ) with a carbide bur in a high-speed handpiece to achieve a length of 14±1 mm in all the samples. The working length was determined with a #15 K-Flexofile (Dentsply Maillefer, Ballaigues, Switzerland), 1 mm away from the apical foramen. 

To prepare the root canals, the coronal thirds of the root canals were enlarged with #4, #3 and #2 Gates-Glidden drills (Mani, Tochigi, Japan), followed by the use of 40/0.10, 35/0.08 and 30/0.06 RaCe rotary files (FKG Dentaire, La-Chaux-de-Fonds, Switzerland) for final preparation of the root canals. The size of master apical file was established at #35. Five mL of 2.5% NaOCl (Taj Corp, Tehran, IRI) was used for irrigation. Syringe delivery was used as irrigation method and the irrigation needle was inserted 4-5 mm into the root canal. After root canal preparation procedures, 1 mL of 17% ethylenediaminetetraacetic acid (EDTA, Pulpdent Corp, Watertown, MA, USA) was used for 5 min to remove the smear layer, followed by 1 mL of 5% NaOCl for 5 min. Finally, the apical foramen was sealed with flowable composite resin (Tetric flow, Ivoclar Vivadent, Schaan, FL, Liechtenstein) and a bonding agent. 

**Figure 1:**
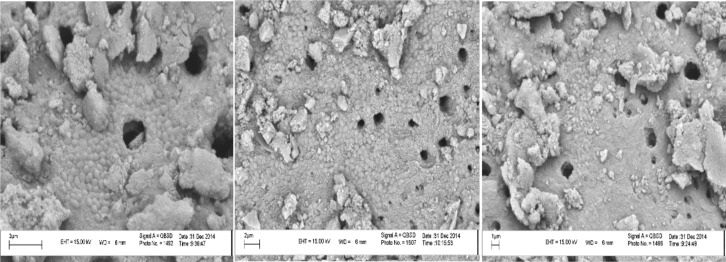
SEM micrographs of transverse sections of teeth to confirm the formation of *E. faecalis*

**Figure 2:**
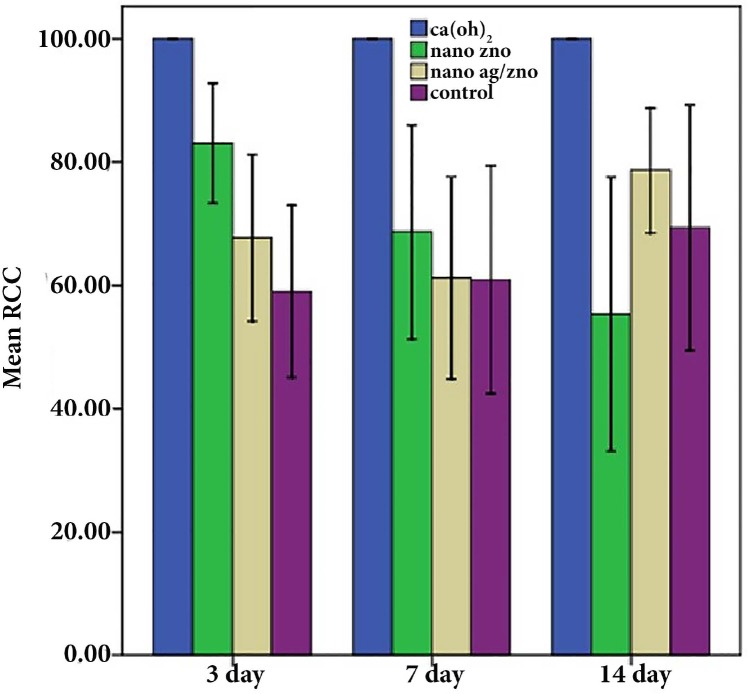
Effect of different antibacterial agents at different time intervals in the experimental and control groups

In order to eliminate bacteria from the culture media, the samples were placed in an autoclave at 121^°^C under 15 PSI for 20 min. The samples were incubated in brain-heart infusion broth (BHI, Merck, Darmstadt, Germany) at 37^°^C for 24 h to confirm sterilization. Then the root canals were filled with a sterile culture medium, incubated at 37^°^C for 2 days and samples were harvested from each root canal in order to verify the sterilization of the samples. In order to prevent the seepage of culture media, the roots were coated with nail varnish. After all the procedures above, the samples were placed within sterile micro tubes in order to carry out the rest of the procedures under more controlled conditions to decrease the odds of bacterial contamination. 

***Bacterial culture and transfer into the samples***

Before initiating the study, the frozen bacteria (-20^°^C) were defrosted and incubated on a solid brain-heart infusion agar plate enriched with 7% sheep blood (Merck, Darmstadt, Germany) at 37^°^C under aerobic conditions. The grown colonies were collected and cultured in Mueller‒Hinton nutrient broth (Merck, Darmstadt, Germany) and again incubated at 37^°^C for 24 h under aerobic conditions. Then a spectrophotometer was used to calibrate *E. faecalis* culture in Mueller‒Hinton broth at 2.5×10^8^ CFUs/mL. A total of 200 µL of the bacterial culture was placed within the lumen of the prepared root canal with a micropipette. In order to make sure of the suitability of conditions to culture bacteria during the procedures, a small amount of the brain-heart infusion (BHI) broth was placed in the coronal area. Then the samples were incubated at 37^°^C for 21 days. After 21 days, the formation of *E. faecalis* in the root canals was confirmed using scanning electron microscope (SEM) (Figure 1).

***Initial bacterial counts***

To determine the initial bacterial counts, normal saline solution was injected into the canal lumen using an insulin syringe to completely fill the root canal. Then a #40 Hedström file (Dentsply Maillefer, Ballaigues, Switzerland) was used 1 mm short of the working length in order to scrape the dentin wall of the root canal to take a sample from the root canal biofilm. Then 10 μg of dentin shavings from each canal was collected. A #50 paper point was placed within the root canal for 60 sec and then transferred into a test tube containing 0.1 mL of sterile normal saline solution as a sampling procedure. Then the test tube was placed on a vortex mixer for 60 sec and a solution with a dilution of 1:10^7^ was prepared from the resultant solution; 0.1 mL of this solution was transferred to blood agar culture media containing sheep blood. The culture media were incubated at 37^°^C for 24 h. Any bacterial growth was evaluated by counting the colonies. These procedures were repeated for all the samples before the use of the intracanal dressing in order to evaluate bacterial contamination and determine the initial bacterial counts. 

***Application of intracanal dressing***

Four dressing materials were used at different time intervals: calcium hydroxide (Prevest Denpro, Golchai, Iran) mixed with 0.12% CHX (Paksan, Tehran, Iran); gel containing ZO nanoparticles (50 ppm); a gel containing nanoparticles of ZO (50 ppm) and sliver (1 ppm); and normal saline solution (the negative control group). In group 1, calcium hydroxide powder was mixed with 0.12% CHX and carried into the canal using lentulo spirals (Mani Inc, Tachigi-ken, Japan) and was packed with a hand plugger (LM Dental, Finland). In groups 2 and 3, the same instruments were used for carrying and packing the gels. Normal saline solution was injected into the root canal at a volume of 0.1 mL using an insulin syringe. The gels containing nanoparticles of ZO and ZO/silver were prepared in Pharmacokinetics Department of Pharmacy Faculty of Tabriz University of Medical Sciences. In group 1, the amount of medicament placed inside the root canal was determined by filling the root canal through the visual assessment and in groups 2 and 3, 30-35 mm^3^ of gels were used for canal dressing. 

A total of 132 prepared samples were randomly divided into 4 main groups (33 samples for each group) using the Rand List software program (with a sampling code of 1314568329) after the initial colony counting procedures. In each group the samples were divided into 3 subgroups (11 samples in each subgroup a, b, c, incubated for 3, 7 and 14 days, respectively) and incubated separately for 3, 7 and 14 days.

All the samples were incubated at 37^°^C for the predetermined durations. After the procedures, the medicaments removed from the root canals by #35 Hedstrom files, 1 mm away from the apical foramen and irrigating with normal saline. 

After incubation procedures for each subgroup, the samples were opened in a sterile environment; samples and bacterial counts were determined in a manner similar to the initial bacterial count determination procedures.

The antibacterial properties of the materials were determined by calculating the percentage reductions in the colony counts (RCC %) in the first and second stages using the formula [5, 8]: (*Initial colony-final colony count*)/*Initial colony count*, where the *initial colony count* was calculated as the bacterial colony count before the use of the intracanal dressing and the *final colony count* was the bacterial colony count after the use of the intracanal dressing.

***Statistical analysis of data***

Data were analyzed with descriptive statistics (means and standard deviations) and one-way ANOVA by considering the effect of time factor on the dependent variable using SPSS17 (Statistical Package for Social Science ,SPSS, version 17.0, SPSS, Chicago, IL,USA). Normal distribution of data was evaluated with Kolmogorov‒Smirnov test. Statistical significance was set at 0.05. 

**Table1:** Mean (SD) initial colony count (ICC) and final colony count (FCC) of each group in different time intervals

**Group**	**Day 3**		**Day 7**		**Day 14**	
	**ICC**	**FCC**	**ICC**	**FCC**	**ICC**	**FCC**
**Calcium hydroxide/CHX**	254.09 (179.75)	0 (0)	210.55 (145.43)	0 (0)	252.36 (345.20)	0 (0)
**Zinc oxide nanoparticles**	257.91 (190.56)	36.82 (51.87)	368.36 (339.53)	143.73 (221.38)	317.45 (216.79)	128.36 (104.19)
**Zinc oxide/silver nanoparticles**	214.27 (178.53)	60.36 (41.17)	442.27 (268.27)	161.27 (99.00)	557.73 (526.62)	88.18 (92.56)

*I.C.C=Initial Colony Count; F.C.C= Final Colony Count)*

**Table 2:** Mean (SD) percentages of reductions in bacterial counts with the use of different dressing agents for different intervals

**Group**	**Day** ** 3**	**Day 7**	**Day 14**
**Calcium hydroxide/CHX**	94.87 (5.35)	87.37 (7.44)	86.73 (10.13)
**Zinc oxide nanoparticles**	83.00 (14.54)	68.71 (25.85)	55.32 (33.05)
**Zinc oxide/silver nanoparticles**	67.73 (20.05)	61.23 (24.34)	78.63 (15.10)
**Control **	42.70 (10.73)	37.83 (11.80)	49.35 (11.13)

## Results

Tables 1 and 2 present the mean initial colony count and final colony count and mean percentages of reductions in bacterial counts with the use of different dressing agents for different time intervals, respectively. Based on the findings, there were no statistically significant differences among mean RCC% of different time intervals with each intracanal dressing agent (*P*=0.09). However, the differences in mean RCC% of different dressing materials were significant (*P*<0.001).

Figure 2 shows the effect of different antibacterial agents at different time intervals in the experimental and control groups. Comparison of the antibacterial effects of experimental agents at different time intervals showed that the mean RCC% of calcium hydroxide in group 1 was higher than those of ZO and ZO/silver nanoparticles and the control group (*P*<0.001). The difference in the antibacterial effect between the ZO and ZO/silver nanoparticle groups was not significant (*P*>0.05). The minimum antibacterial effect was observed in the control group (*P*<0.0001). 

## Discussion

Different materials have been used as intracanal dressing, but none of them has been shown the properties of ideal material. Several researches in order to introduce material with better properties and overcoming the bacterial resistance against antibiotics have been resulted in application of nano technology in production of antimicrobial agents including ZO and silver nanoparticles [11,14]. In this *in vitro* study, we compared the antibacterial effects of gels containing ZO and ZO/silver nanoparticles and a mixture of calcium hydroxide and 0.12% CXH as intracanal medicaments in root canals contaminated with *E. faecalis* at different time intervals.

Based on the results of the present study, a mixture of calcium hydroxide and CHX was completely effective against *E. faecalis*, *i.e.* mixing calcium hydroxide with CHX was beneficial and significantly increased the antibacterial effect of this material on *E. faecalis*, consistent with the majority of previous studies [19-22]. Delgato *et al.* [23] compared the antibacterial effects of calcium hydroxide and CHX as intracanal medicaments on *E. faecalis* and reported that CHX had a stronger antibacterial effect compared to calcium hydroxide and incorporation of calcium hydroxide into chlorhexidine did not increase its antibacterial activity. However, incorporation of CHX into calcium hydroxide increased its antibacterial activity. On the other hand, contrary to the majority of previous studies [19-22], the results of a study by Balal *et al*. [24] on the effect of adding other materials to calcium hydroxide showed that adding CHX to calcium hydroxide was ineffective and such a mixture did not increase the antibacterial activity of calcium hydroxide. In addition, such mixture decreased the antibacterial activity of CHX. Although the study above showed that adding CHX to calcium hydroxide was ineffective, it should be pointed out that such mixing has been carried out to improve the antibacterial effects of the most commonly used intracanal medicament and is cost-effective from an economic viewpoint. Moreover, the conflicting results of the studies evaluating the antibacterial activity of calcium hydroxide/CHX combination could be attributed to different sample sizes, various amounts of CHX and calcium hydroxide in the mixture and differing method and time intervals of assessments [23, 24].

Use of ZO and ZO/silver nanoparticles in the present study were consistent with efforts made in other studies to overcome the disadvantages of the currently used materials and introduce a material with proper antibacterial activity and biocompatibility properties. Analysis of the results of antibacterial effects of these nanoparticles and comparison of these properties with those of calcium hydroxide/CHX showed that they were less effective than the mixture of calcium hydroxide and CHX. In this context, zinc oxide and zinc oxide/silver nanoparticles destroyed 69.01% and 69.19% of bacteria, respectively, but the mixture of calcium hydroxide and CHX destroyed 89.65% of bacteria. By considering the similarity between the effect of calcium hydroxide/CHX mixture used in the present study and other studies [19-22], the low antibacterial effect of nanoparticles used in the present study might be attributed to the low available concentration of these materials in the vicinity of walls contaminated with bacteria. In the present study, based on the concentration suggested in previous studies [16, 25] and by considering biocompatibility and cytotoxicity issues, 50 ppm and 1 ppm concentrations of ZO and ZO/silver (in the form of silver nitrate nanoparticles), respectively, were used as intracanal medicaments in the form of gels. Gel forms were used in the present study to make the materials remain within the root canal and prevent their diffusion to the tissues beyond the root apex. However, the results showed that such a technique might decrease the available material in the vicinity of root canal walls, which explains the lower antibacterial effect of these materials in comparison to the mixture of calcium hydroxide/CHX in the present study. However, based on the results, forms other than gels cannot be used for such mixtures because if the material’s flow rate increases (for better diffusion within the root canal space), the odds of hypersensitivity reactions in the periradicular tissues will increase [25, 26]. 

The results of various studies show that the antibacterial activity of nanoparticles depends on the concentration and duration of their contact [26, 27]. Therefore, higher concentrations can be used for more antibacterial effects. In this context, in a study by Mandal *et al*. [26], use of a gel containing silver nanoparticles at a concentration of 0.02% gave rise to better results compared to calcium hydroxide; however, there are concerns in relation to biological aspects. 

In addition, based on the results of the present study, there were no significant differences in the antibacterial effects (RCC%) at different intervals of 3, 7 and 14 days. The time intervals were sleeted based on time intervals during clinical procedures. Although in previous studies no exact times have been reported for the application of calcium hydroxide (alone or in combination with other antibacterial agents) to achieve antibacterial properties [9, 28], some studies have not recommended applications for less than 14 days [29]. In this study, the incubation period of 3 days was used according to the similar studies [7, 20, 30, 31]. Also Pavaskar *et al.* [30] concluded that calcium hydroxide effectively inhibits *E. faecalis* within dentinal tubules up to 72 h. The results of the present study in this context are consistent with the results of previous studies. In a study by Sjögren *et al.* [32] on the antibacterial effects of short-term use of calcium hydroxide, a minimum of 7 days was recommended for maximum antibacterial activity. Based on the results of that study, a 10-min application destroyed only a small number of bacteria; however, longer application (for more than 24 h) resulted in the elimination of *E. faecalis* species. The results of the present study showed the effect of a similar intracanal medicament, *i.e.* calcium hydroxide/CHX, at a time interval longer than 24 h similar to what was suggested by Sjögren *et al*. [32]. In addition, some studies have not suggested application of less than 14 days for intracanal dressings [29]. The results of the present study confirmed this to some extent because in none of the used materials, a 100% antibacterial effect was achieved with the use of intracanal dressings for less than14 days. 

One of the limitations of the study was the low antibacterial effect of nanoparticles in the form of gel; considering the biocompatibility considerations, other forms of intracanal medicaments such as paste or liquid should be used in the future studies. Also future studies are needed with higher sample size and longer time periods of intracanal medicaments. 

## Conclusion

The mixture of calcium hydroxide/CHX showed the highest antibacterial effect. Also the antibacterial activity of ZO was not improved by adding silver nanoparticles. In addition, there were no significant differences in the antibacterial effects of intracanal medicaments at 3-, 7- and 14-day intervals. 
